# Alpha lipoic acid antagonizes cytotoxicity of cobalt nanoparticles by inhibiting ferroptosis-like cell death

**DOI:** 10.1186/s12951-020-00700-8

**Published:** 2020-10-02

**Authors:** Yake Liu, Wenfeng Zhu, Dalong Ni, Zihua Zhou, Jin-hua Gu, Weinan Zhang, Huanjian Sun, Fan Liu

**Affiliations:** 1 Department of Orthopaedics, Affiliated Hospital of Nantong University, 20 Xisi Road, Nantong, 226001 Jiangsu China; 2 Orthopaedic Laboratory, Affiliated Hospital of Nantong University, Nantong, Jiangsu Province China; 3 Department of Radiology, University of Wisconsin-Madison, 11111 Highland Avenue, Madison, WI 53705 USA; 4 Department of Clinical Pharmacy, Affiliated Maternity and Child Health Care Hospital of Nantong University, Nantong, Jiangsu Province China; 5 Department of Orthopaedics, The Sixth Affiliated Hospital of Nantong University, Yancheng, Jiangsu Province China

**Keywords:** Cobalt nanoparticles, Nanotoxicity, Ferroptosis, Alpha-lipoic acid, Detoxification

## Abstract

As a main element in the hard metal industry, cobalt is one of the major components of human metal implants. Cobalt-containing implants, especially joint prostheses used for artificial joint replacement, can be corroded due to the complex physiological environment in vivo, producing a large number of nanoscale cobalt particles (Cobalt Nanoparticles, CoNPs). These CoNPs can be first accumulated around the implant to cause adverse local reactions and then enter into the blood vessels followed by reaching the liver, heart, brain, kidney, and other organs through systematic circulation, which leads to multi-system toxicity symptoms. To ensure the long-term existence of cobalt-containing implants in the body, it is urgently required to find out a safe and effective detoxification drug. Herein, we have demonstrated that CoNPs could induce the ferroptosis-like cell death through the enhancement of intracellular reactive oxygen species (ROS) level, cytoplasmic Fe^2+^ level, lipid peroxidation, and consumption of reduced glutathione (GSH) as well as inhibition of glutathione peroxidase 4 (GPX4) activity. Importantly, α-lipoic acid (ALA), a natural antioxidant with the capability to scavenge free radicals and chelate toxic metals, was found to efficiently alleviate the adverse effects of CoNPs. The present study illustrates a new mechanism of CoNPs mediated by ferroptosis-like cytotoxicity and discloses an effective method for the detoxification of CoNPs by employing the natural antioxidant of ALA, providing a basis for further in vivo detoxification study.
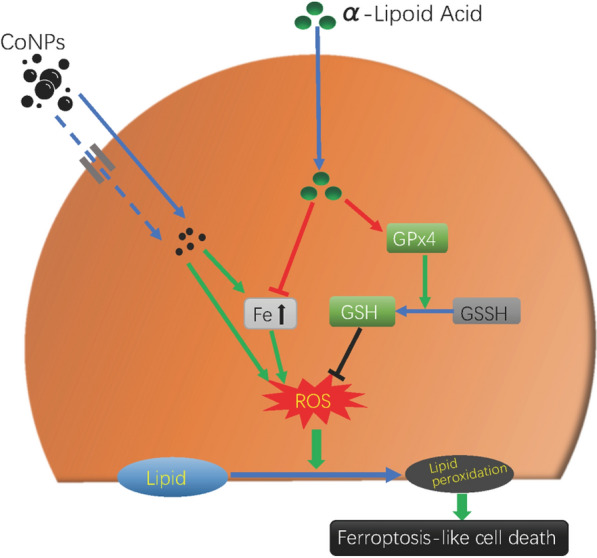

## Introduction

Hard metal is an alloy based on tungsten carbide (WC) and cobalt (Co), which is the key component of the joint prosthesis for artificial joint replacement. When an artificial joint is implanted in the body, degradation of the prosthesis is inevitable due to different physical and chemical effects such as mechanical stress, fretting, or fatigue corrosion, leading to the generation of metal particles with different diameters. Among these particles, cobalt nanoparticles (CoNPs) are the most abundant ones with the highest toxicity [1, 2], which could cause early failure of artificial joints [3]. Tower et al*.* well demonstrated the multiple systemic adverse reactions after metal-on-metal (MOM) joint replacement and named the disease as arthroprosthetic cobaltism syndrome (ACS) [4–8]. CoNPs produced by the reduction or wear of internal plants are first deposited around the internal fixation plate and artificial joint prosthesis to cause inflammatory pseudotumor around the implant, loosening of the prosthesis, as well as local osteoporosis. Meanwhile, due to their nano-size effect, CoNPs are more likely to enter into the blood and lymphatic system, followed by their broad distribution in multiple organs or tissues of the human body through systemic circulation, resulting in different adverse symptoms [4–6, 8–11]. Therefore, the patients undergoing MOM joint replacement need to be monitored and detoxified. It is urgent to investigate the detailed toxicity mechanism of CoNPs thoroughly and identify safe and effective antidote drugs, which is of great significance but has not been reported yet.

After the internalization of CoNPs into the cytoplasm through endocytosis, the nanoparticles can induce the decomposition of H_2_O_2_ into more toxic HO^.^ radicals through a Fenton-like reaction and produce a large number of reactive oxygen species (ROS)[12]. Oxidative stress is found to be one of the key mechanisms of cobalt cytotoxicity [1, 13–17]. However, the molecular mechanism of oxidative stress-induced cell death of CoNPs remains unclear. The clinical treatment for the cobalt-related toxic symptoms is mainly to reduce the plasma cobalt concentration through various means including prosthesis and implant removal, plasma exchange [18, 19], chelating agent EDTA [20, 21], and dimercapto propanol sulfonic acid sodium [22]. However, all these strategies failed to exhibit the desired therapeutic effect [23]. For the treatment of systemic and local toxicity symptoms, no safe and effective therapeutic drugs have been reported until now.

Lipoic acid is an essential cofactor for mitochondrial metabolism and is required for the catalysis of various mitochondrial 2-keto acid dehydrogenase complexes. It plays an essential role in stabilizing and regulating these multienzyme complexes. Moreover, it is responsible for cellular growth and mitochondrial activity as well as necessary for the coordination of energy metabolism [24]. In the latest generation of antioxidants, lipoic acid can alleviate the hydroxyl radical, hypochlorous acid, singlet oxygen, and peroxy radicals [25]. Besides, they can chelate iron, copper, and other transition metals [26] by increasing intracellular reduced glutathione (GSH) levels [24]. It is worth investigating the therapeutic effect of lipoic acid in Ameliorating the toxicity caused by the CoNPs.

Herein, we found the mechanism of ferroptosis-like mediated cytotoxicity of CoNPs and further disclosed a new method for the detoxification of CoNPs by employing the natural antioxidant of ALA. The Balb/3T3 cells were incubated with CoNPs and α-lipoic acid (ALA) under different conditions. The results demonstrated that CoNPs could induce the elevation of the levels of Fe^2+^ ion concentration, ROS, and lipid peroxidation. Moreover, the intracellular GSH was excessively consumed, and glutathione peroxidase-4 (GPx4) activity was greatly inhibited, leading to ferroptosis-like cell death. Importantly, alpha-lipoic acid can antagonize the ferroptosis-like toxicity of CoNPs in Balb/3T3 cells. Overall, this study reveals new insight into the mechanism of cobalt toxicity and provides a new method of detoxification by employing ALA.

## Results

### Cell viability assay

**Fig. 1:**
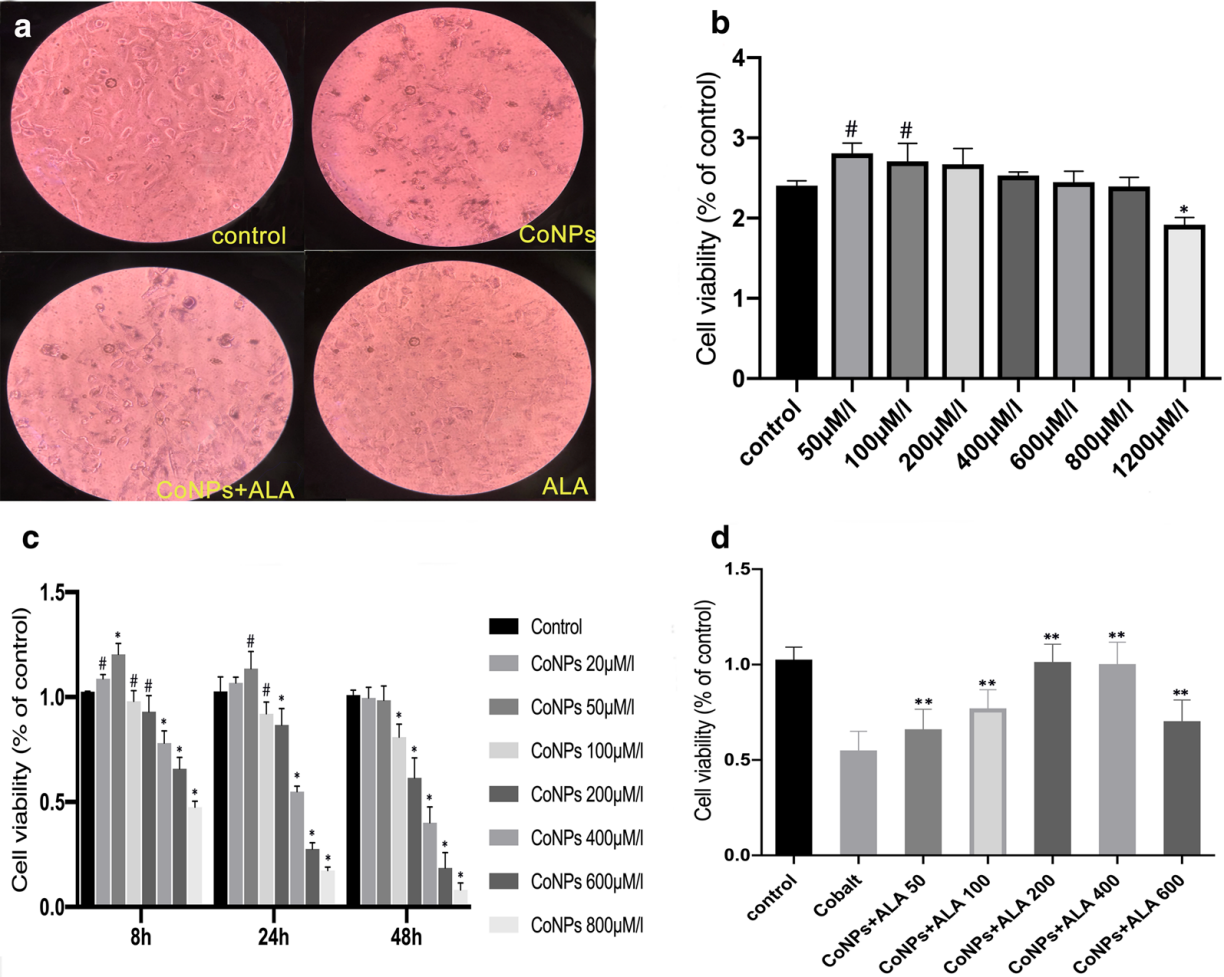
CoNPs induced Balb/3T3 cell death, while ALA significantly inhibited its cytotoxicity. **a** The cells were incubated with CoNPs (400 μM) and ALA (200 μM) for 24 h. The cell morphology became round with smaller volume and the cell nucleus was concentrated in response to CoNPs treatment. Interestingly, ALA could reverse these changes induced by CoNPs(40X). **b** ALA exhibited low cytotoxicity toward Balb/3T3 cells, and it could significantly reduce cell activity only when its concentration is greater than 1200 μM. **c** CoNPs induced cytotoxicity in both dose- and time-dependent manner. **d** ALA could reverse the cytotoxicity induced by CoNPs. All the data were expressed as mean ± SD of three independent experiments performed in triplicates. (*p < 0.01, #p < 0.05 vs. control, **p < 0.01 vs. cobalt group)

Balb/3T3 cells were exposed to varying concentrations of CoNPs for 6, 24, and 48 h, and the cell viability assay was determined using the CCK-8 assay kit. The results showed that CoNPs treatment inhibited the proliferation of Balb/3T3 cells in a dose- and time-dependent manner. When the concentration was less than 200 μM or exposure time was less than 24 h (Fig. 1c), CoNPs could slightly stimulate cell proliferation. There was no significant decrease in cell viability for the treatment of 6 h. However, there was around 50–60% reduction of viability of cells incubated with 400 μM of CoNPs for 24 h (Fig. 1a, c). Therefore, subsequent experiments were performed with 400 μM of CoNPs. It was observed that the low and medium concentrations of ALA could promote cell proliferation slightly. In contrast, the cell viability was significantly inhibited at higher concentrations (> 200 μM) of ALA (Fig. 1b). Interestingly, the co-incubation of CoNPs (400 μM) and ALA at different concentrations for 24 h showed that the CoNPs-induced cytotoxicity was greatly inhibited by ALA in a dose-dependent manner (Fig. 1d). Besides, we stained the cells with Calcein AM/PI after 24 h of exposure to cobalt nanoparticles and lipoic acid. The results of fluorescence microscope observation showed that 400 μM CoNPs caused significant changes in cell viability, with smaller cell sizes, more dead cells than live cells, and ALA significantly antagonized cell death caused by CoNPs (Additional file 1: Figure S3).

### Determination of intracellular ROS and MDA levels

**Fig. 2:**
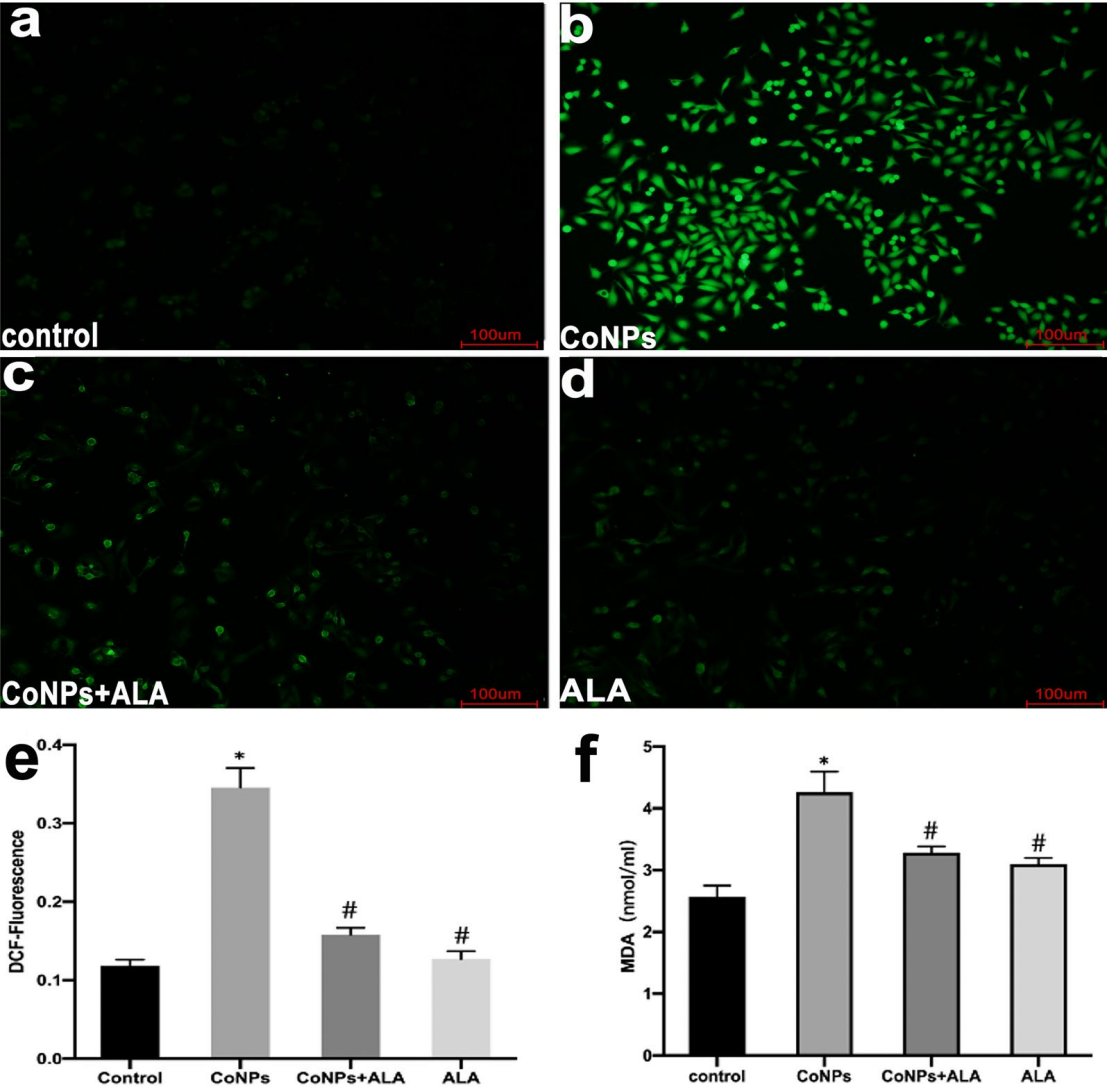
CoNPs treatment enhanced the level of intracellular ROS in Balb/3T3 cells, while ALA significantly inhibited the formation of ROS. The cells were exposed to CoNPs (400 μM) and ALA (200 μM) for 24 h. **a**–**d** The cells were visualized using a confocal laser scanning microscope. Each slide was scanned at ×20. The green fluorescence indicates the detected ROS production. **e** The fluorescent intensity of cells with different treatments. **f** The intracellular MDA concentration of cells with different treatments. All the data were expressed as mean ± SD of three independent experiments performed in triplicates. *p < 0.01, #p < 0.05 vs. control

Our experimental results showed that CoNPs treatment (400 µM, 24 h) induced a significant increase in ROS level of Balb/3T3 cells as compared to the control experiment, suggesting that the formation of ROS was one of the plausible mechanisms of CoNPs-mediated cell death. Besides, the CoNPs-induced formation of intracellular ROS was significantly inhibited when incubating CoNPs with ALA (200 µM), showing the important roles of ALA in scavenging ROS (Fig. 2a–e). Spectrophotometric results further revealed that CoNPs could elevate the level of cellular MDA, but it could be antagonized by ALA (Fig. 2 f).

### Apoptosis assay in Balb/3T3 cells

**Fig. 3:**
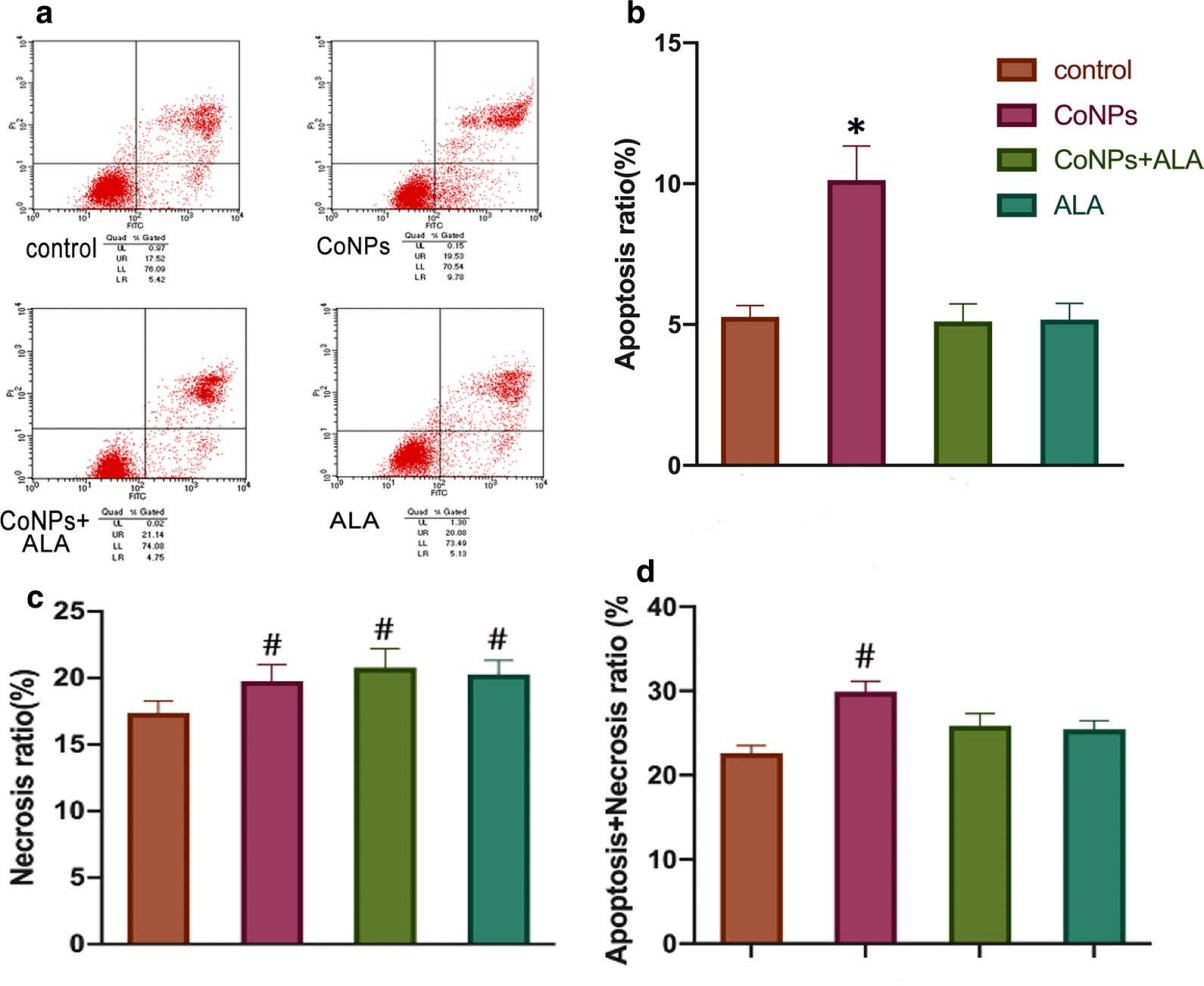
Cell apoptosis and necrosis levels of Balb/3T3 cells in presence of CoNPs and ALA. The cells were co-incubated with CoNPs (400 μM) and ALA (200 μM) for 24 h. Flow cytometry results exhibited that CoNPs induced mild cellular apoptosis, which could be further alleviated by the exposure of ALA. The apoptosis rate for the CoNPs treatment group was significantly different as compared to the control group. After 24 h of CoNPs exposure, the sum of apoptosis rate and necrosis rate was also significantly higher than of the control group, but the increment was lower than the results of the cell viability experiment (CCK-8 assay, Fig. 1c). All the data were expressed as mean ± SD of three independent experiments performed in triplicates. *p < 0.01, #p < 0.05 vs. control

Flow cytometry results in Balb/3T3 cells showed that the apoptosis rate of cells treated with CoNPs (9.78%) was increased slightly as compared to that of the control group (5.42%). The ALA could also slightly alleviate the CoNPs-induced cell apoptosis (4.75%) (Fig. 3). It is worth mentioning that the apoptosis rate induced by CoNPs (9.78%) was significantly lower than the corresponding cell death rate (45%), as observed by the CCK-8 assay (Figs. 3d and 1c).

### Determination of intracellular iron, cobalt concentration in Balb/3T3 cells

**Fig. 4:**
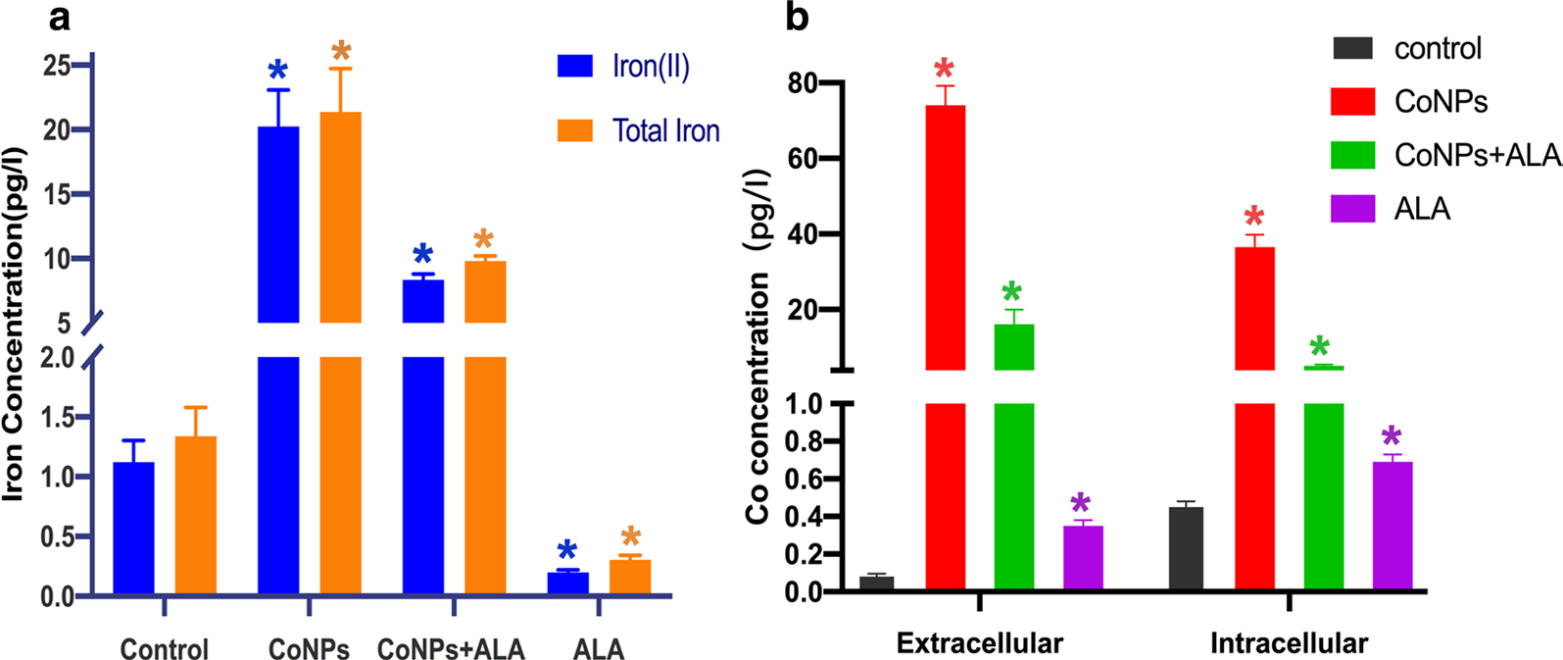
Iron and cobalt concentration. **a** Spectrophotometry based determination of intracellular iron concentration in Balb/3T3 cells. The intracellular Fe^2+^ ion and total iron (Fe^2+^ + Fe^3+^) concentration of Balb/3T3 cells with different treatments. All the data were expressed as mean ± SD of three independent experiments performed in triplicates. *p < 0.01 vs. control. **b** ICP-MS measurement of intracellular &extracellular cobalt concentration in Balb/3T3 cells. Compared with the control group, the concentration of cobalt inside and outside the cells in the CoNPs-treated group was significantly increased, and ALA had a significant antagonistic effect on this. Note: In this figure, the intracellular and extracellular cobalt concentrations are not comparable due to the different processing methods of the cell. All the data were expressed as mean ± SD of three independent experiments performed in triplicates. *p < 0.01 vs. control

The intracellular iron concentration was further performed and the spectrophotometry results illustrated that CoNPs treatment induced a significant increment of Fe^2+^ ion and total iron concentration in Balb/3T3 cells as compared to the control group. However, ALA exhibited a significant antagonistic effect on CoNPs induced enhancement of intracellular Fe^2+^ ion and total iron concentration upon co-exposure of CoNPs and ALA toward cells. ALA treatment alone did not promote any increment of intracellular Fe^2+^ ion and total iron concentration (Fig. 4a). After the cells were exposed to CoNPs for 24 h, compared with the control group, the intracellular and extracellular cobalt concentration of the CoNPs-exposure group increased significantly, and ALA had a significant antagonistic effect on this. From the results, we can speculate that ALA can inhibit the uptake of CoNPs by cells or promote the excretion of CoNPs to reduce the intracellular Co concentration, and inhibit the cells from converting CoNPs into cobalt ions (Fig. 4b).

### Measurement of intracellular GSH in Balb/3T3 cells

**Fig. 5:**
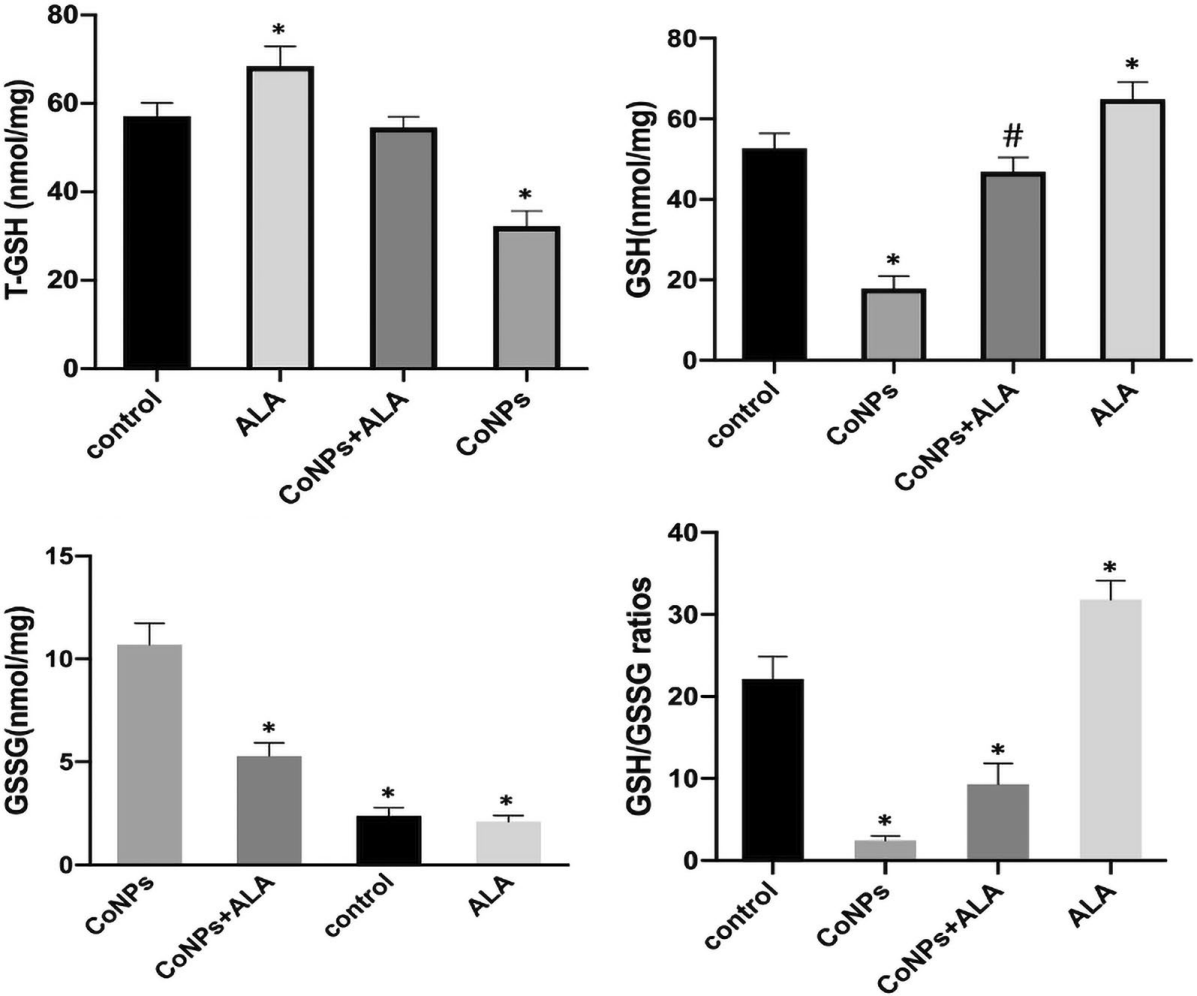
Spectrophotometric measurement of intracellular reduced and oxidized GSH in Balb/3T3 cells. The cells were exposed to CoNPs (400 μM) and ALA (200 μM) for 24 h. The administration of CoNPs led to a reduction of the levels of T-GSH, GSH, and GSH/GSSG ratio, as well as the elevation of the GSSG level. Importantly, all the CoNPs-induced effects were alleviated by ALA significantly for the co-treatment group. All the data were expressed as mean ± SD of three independent experiments performed in triplicates. *p < 0.01, #p < 0.05 vs. control

GSH is the key cellular endogenous antioxidant that can scavenge toxic free radicals, produced in response to various causes of oxidative stress, thereby relieving the oxidative damage. It should be mentioned here that the ferroptosis-like cell death is related to GSH depletion. To comprehend whether CoNPs could cause ferroptosis-like cell death, Balb/3T3 cells were exposed to CoNPs alone, ALA alone as well as CoNPs along with ALA for 24 h, and the corresponding cell lysates were subjected to spectrophotometric measurement of total GSH, GSH, and GSSG. It was found that CoNPs can significantly reduce the intracellular level of total GSH, GSH, and GSG/GSSH ratio as compared to the control group. The cells in the co-exposure group (CoNPs + ALA) did not exhibit any decrease in the level of total GSH, GSH, and GSSG. The results altogether suggested that CoNPs treatment reduced the cell’s antioxidant function by depleting the intracellular level of GSH, while ALA exhibited an effect of stabilizing the cell’s antioxidant function (Fig. 5, Additional file 1: Table S1).

### Western blot analysis for checking the intracellular level of GPx4

**Fig. 6:**
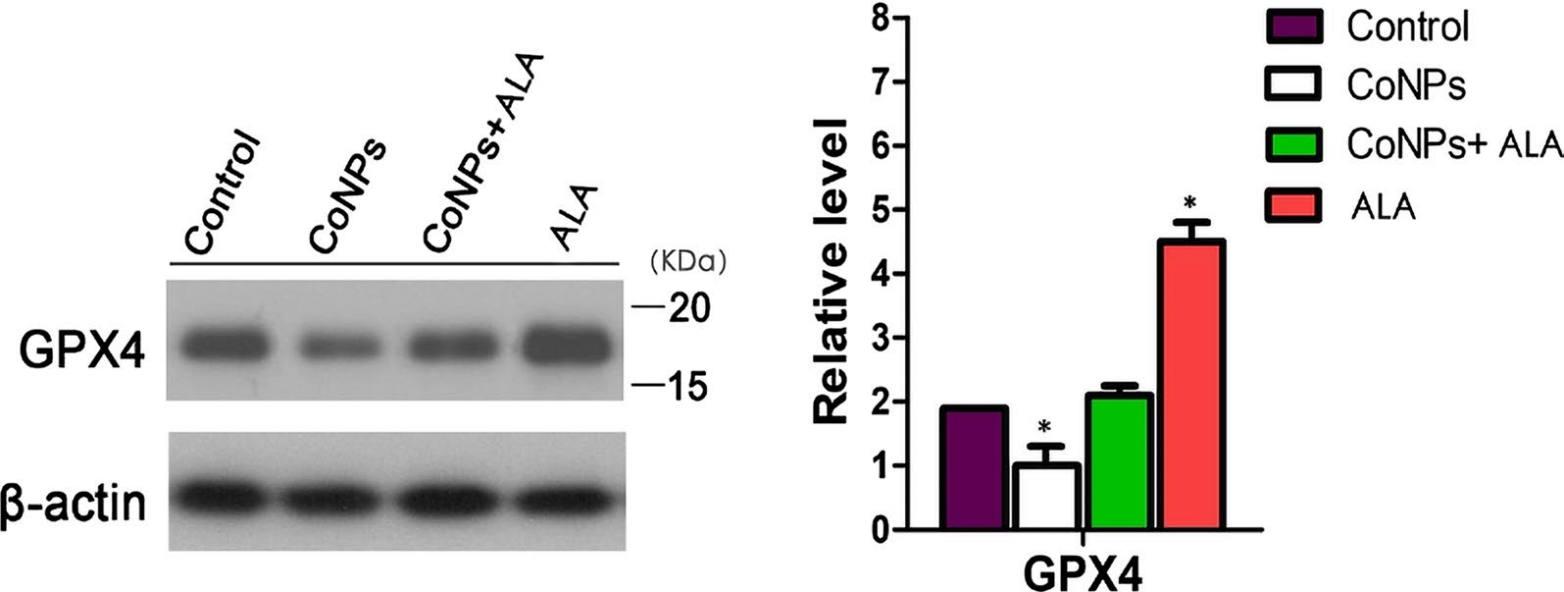
Western blot analysis in Balb/3T3 cells. The results exhibited that the administration of CoNPs led to a lower expression of GPx4. However, ALA could significantly relieve the decrease of GPx4 expression induced by the CoNPs when the cells were exposed with both CoNPs and ALA. *p < 0.01, vs. control

The western blot analysis in Balb/3T3 cells showed that CoNPs treatment inhibited the expression of GPx4 significantly as compared to the control group. In contrast, the co-exposure group (CoNPs + ALA) exhibited a significant increment of GPx4 level. When the cells were treated with ALA alone, the GPx4 expression was significantly increased in comparison with the control group. Overall, western blot analysis indicated that ALA could reverse the effect of CoNPs on the GPx4 level (Fig. 6).

### Transmission electron microscope observation of cell structure changes

**Fig. 7:**
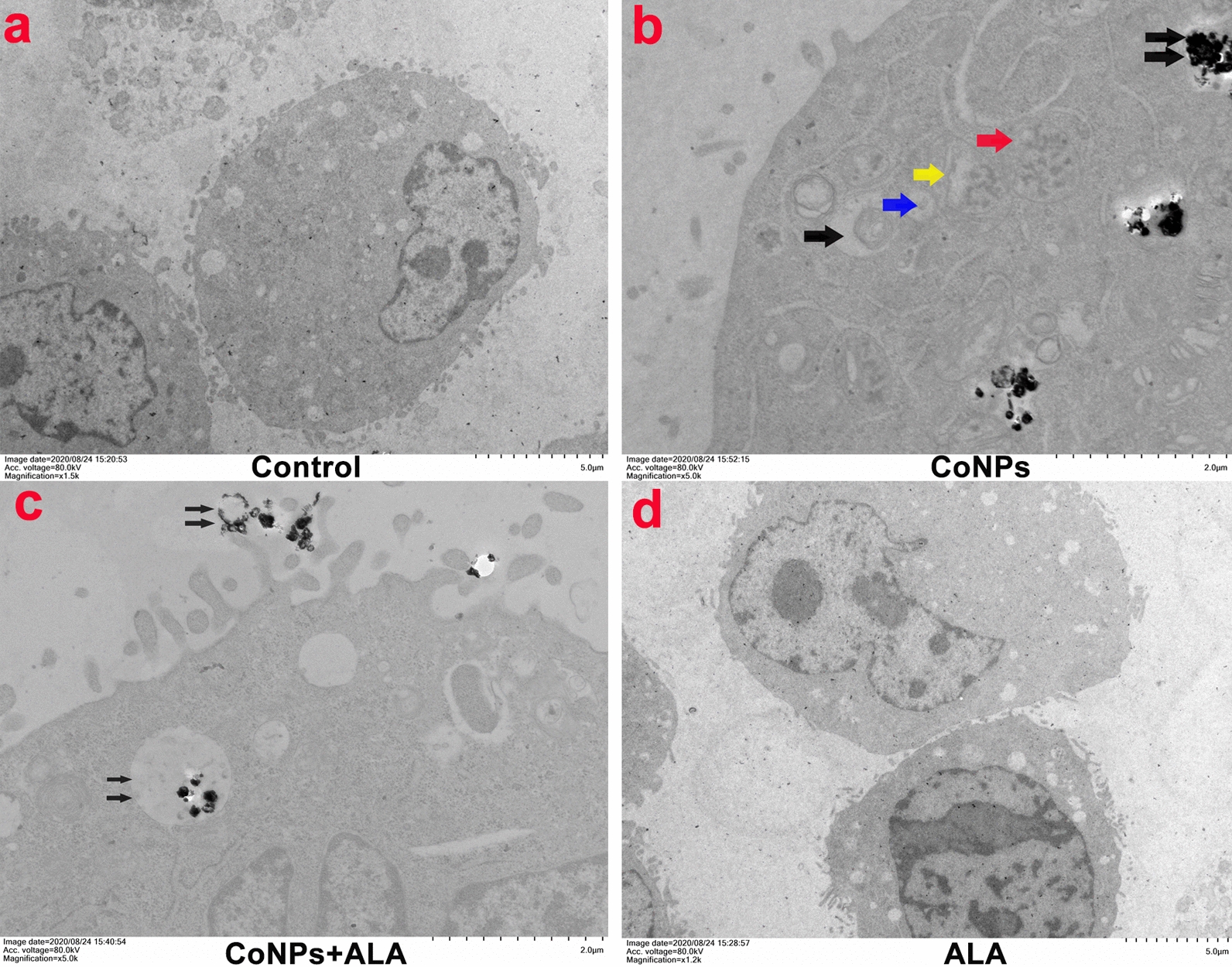
TEM image of cells treated with CoNPs and ALA for 24 h. **a** Control group. No abnormality in cell morphology and structure was found; b) Morphological characteristics of CoNPs. The CoNPs are round or oval, with size less than 50 nm; **b** Cells treated with CoNPs only group. Obvious characteristic changes in mitochondria can be found. The mitochondria shrunken, while the mitochondrial membrane is visible. The chromatin is condensed in granular form (red arrow). The blue arrow indicates the early stage of mitochondrial deformation, while the yellow arrow indicates the middle stage of mitochondrial deformation. Intracellular organelles are swollen and have many vesicle shapes (black arrow). CoNPs are aggregated and encapsulated in the cell (double black arrow); **c** Cells treated with CoNPs and ALA together, CoNPs inside the cells are wrapped to form vesicles (double black arrow) and are excreted from the cell. The cell morphology and structure are similar to those of the control group. **d** ALA group, the cell morphology, and structure are similar to those of the control group

Obvious characteristic changes in mitochondria with cells treated with CoNPs only could be found. The mitochondria shrink, but the mitochondrial membrane is visible, and the chromatin is broken and condensed in granular form. The chromatin is concentrated in granular form. Intracellular organelles are swollen and have many vesicle shapes. CoNPs are aggregated and encapsulated in the cell. In contrast, the morphology and structure of cells treated with CoNPs and ALA together are similar to those of the control group, showing negligible damage of cells (Fig. 7, Additional file 1: Figures S2 and S4).

## Discussion

The detailed molecular mechanisms of cytotoxicity and genotoxicity of cobalt as well as cobalt compounds are still unclear. Based on the previous reports, there are some major aspects which are described below: (1) Oxidative stress: CoNPs and cobalt ions can produce excessive ROS through Fenton reaction [27, 28], as well as enhancement of intracellular antioxidant enzyme (e.g. catalase, glutathione peroxidase, and heme oxygenase-1, etc.) to degrade ROS. Harmful biological effects and irreversible damage are generally caused because of the oxidation of proteins, lipids, carbohydrates, and DNA, induced by excessive ROS. At the same time, excessive ROS could promote the release of inflammatory factors, deplete glutathione, and reduce the activity of antioxidant stress-related proteins such as GPx4, superoxide dismutase, catalase, etc. As a result, chromosomal damage of cells, apoptosis, and necrosis is caused through various pathways [28–31]. (2) Hypersensitivity: Cobalt, chromium, and nickel are well-known immunosensitizers. The adverse reactions of MoM hip prosthesis wear particles might be caused by the metal ion hypersensitivity [32, 33]. Earlier studies have demonstrated that patients with MoM hip prostheses have a higher risk of lymphopenia and metal hypersensitivity reactions [34]. In vivo studies have also confirmed that micron-scale rather than nano-scale chromium-nickel particles cause type IV hypersensitivity reactions [35]. (3) Autophagy and endoplasmic reticulum stress: Metal particles generated from the wears often induce stress in the endoplasmic reticulum, mediating the expression of a variety of inflammatory factors (TNF-α, IL-6, and IL-1β). It is found that autophagy is involved in promoting the apoptosis of osteoblasts which are caused by wear particles and inhibits the plasma reticulum stress. Moreover, autophagy can reduce inflammatory factor secretion and osteoclast production and alleviate the effects of wear particles on osteoblasts [36–38]. (4) Destroy cell structure: CoNPs and cobalt ions could directly bind to proteins with multiple functions in the cells including redox system enzymes, metabolism, molecular transport, cell signaling, and organelles [39]. Additionally, they can induce oxidation and the loss of biological function, as well as destroy the skeleton of cells directly, leading to chromosome condensation and aberrations. Cobalt could also bind to several proteins involved in cellular redox systems and active oxygen scavenging. Furthermore, cobalt has also been shown to interact with various receptors, ion channels, and biomolecules [40, 41]. (5) Replace essential metal ions: Earlier report demonstrated that cobalt can interact with various metal-based proteins (e.g. zinc-finger protein, catalase, etc.) and replace the corresponding metal ions (e.g. Mg^2+^, Ca^2+^, Zn^2+^, etc.). Therefore, the functions of these proteins or enzymes are lost or altered [39]. All these previous reports altogether suggest that CoNPs can induce cell death through oxidative stress.

Our study also confirmed that the administration of CoNPs toward Balb/3T3 cells led to enhancement in the intracellular ROS level as compared to the control group (Fig. 2). However, the exact molecular mechanism of how ROS causes cell death remains unclear. Although CoNPs-treatment caused a significant increment of apoptosis rate of Balb/3T3 cell as compared to the control group (9.78% VS. 4.75%) (Fig. 3 a, b, e), it was still much lower than the cell death rate, as observed by the CCK-8 assay under the same conditions (9.78% vs. 45%) (Fig. 1f). These results suggest that non-apoptotic mechanisms are involved in CoNPs-mediated cellular death.

Ferroptosis is a process to regulate cell death, discovered in recent years. It is different from other forms of cell death in terms of cellular morphology and molecular mechanism. It is characterized by the iron-dependent accumulation of lipid hydroperoxide to lethal levels [42, 43]. The main features of ferroptosis are the cellular uptake of iron, the formation of intracellular ROS through the Fenton reaction, excessive consumption of GSH in the cells, and the inactivation of GPx4. These processes ultimately cause lipid peroxidation and production of lethal reactive oxygen species to promote cell death [43]. Ferroptosis is majorly associated with iron ion imbalance, oxidative stress, and GSH homeostasis. Although it has been reported that Ca^2+^ influx and GSH depletion play an important role in ferroptosis [44] and oxidative death (Oxytosis), the specific mechanism by which iron ions induce cell death remains unclear [45].

Ferroptosis has widely been confirmed in different diseases such as tumors and neuropathy and is one of the current research hotspots. Due to the similarity of the physical and chemical properties of cobalt and iron elements, the comparative study of the two elements is also meaningful. The mechanism of cobalt toxicity is highly similar to the characteristics of ferroptosis. The earlier study showed that cobalt treatment led to a significant increment of intracellular ROS, lower GSH level, as well as the activation of the Nrf2 signaling pathway. However, different antioxidants (such as N-acetylcysteine, alpha-Tocopherol, etc.) could exhibit antagonistic effects on cobalt toxicity and iron death [43, 46–56]. Our study reveals that CoNPs could induce the production of excessive ROS in Balb/3T3 cells, overproduction of lipid peroxidation products (MDA), and enhancement of cellular ferrous ion concentration. Moreover, CoNPs-treatment led to the reduction of intracellular GSH level and GPx4 activity. To be interesting, the TEM image shows the characteristic changes of mitochondrial atrophy, but the mitochondrial membrane is clearly visible, and chromatin is broken and granularly condensed in granular form (Fig. 7d). In summary, all these results suggest that CoNPs can activate ferroptosis in Balb/3T3 cells by stimulating oxidative stress, or directly induce ferroptosis-like death. Similar views were also provided by Gupta et al*.* [57] when studying Parkinson’s pathogenesis in neuronal cells.

According to different signaling pathways and molecular mechanisms, the current detoxification drugs for CoNPs toxicity in vitro or in vivo are following categories: (1) Antioxidants such as nitrogen-acetylcysteine (NAC) [50], L-ascorbic acid (LAA) [46], melatonin [58], alpha-tocopher [48], etc. (2) Antagonists, such as zinc [59, 60]; (3) Chelating agents, such as EDTA [20, 21] and dimercaptopropanol sulfonic acid sodium [22]; (4) Endoplasmic reticulum stress inhibitor, such as sodium 4-phenylbutyrate [36, 37]. Based on previous studies, anti-oxidative stress is the key direction of detoxification research. The ideal medicine could be one that can resist oxidative stress and chelate with the metal ions. Moreover, it should be soluble in both water and fat as well as safe in vivo [61]. With this speculation, we discovered that ALA could effectively attenuate the CoNPs induced ferroptosis-like cell death in Balb/3T3 cells.

Lipoid acid (LA) is unique among different natural antioxidants and is a highly effective therapeutic agent associated with oxidative damage [62]. ALA is a dithiol compound that usually binds to the lysine residue of mitochondrial α-keto acid dehydrogenase. It is well-known that cytoplasmic and mitochondrial dehydrogenase can quickly reduce LA into dihydrolipoic acid (DHLA) [63]. Previous reports have demonstrated that ALA could bind iron or any divalent metal. Therefore, it's iron-chelating capability decreases the amount of free iron in the body system, thereby reducing oxidative stress by scavenging free radicals. Moreover, ALA could alleviate cytotoxicity during iron overload through the reduction of glutathione content, mitochondrial dysfunction, as well as gene expression of heme oxygenase-1b and superoxide dismutase [64].

In the latest generation of antioxidants, lipoic acid can scavenge a variety of free radicals including hydroxyl radicals, hypochlorous acid, oxygen singlet, and peroxyl radicals, due to its solubility in both water and fat [25, 65]. On the other hand, conventional antioxidants such as vitamins A, E, C, and selenium can only interact with one or two free radicals [66]. In theory, lipoic acid seems to be the most effective drug among all antioxidants [62]. Additionally, lipoic acid can also interact with GSH and recycle endogenous GSH, stabilizing the antioxidant capacity of cells [64, 67–69]. LA has changed arrangement on its fatty acid-like skeleton, which allows it to capture the copper and iron ions, thereby forming a stable complex with these metals to achieve chelation of iron, copper, cobalt, manganese, zinc, cadmium, lead, nickel, arsenic, mercury and other transition metal ions [26, 62, 69–71]. In the present study, we found that CoNPs significantly induced overproduction of intracellular ROS, enhancement of intracellular iron(II) and total iron concentration (Fig. 4), inhibition of GPx4 activity (Fig. 6), and reduction of reduced intracellular GSH (Fig. 5) as compared to the control group. Importantly, ALA can significantly antagonize the CoNPs induced above adverse results, thereby maintaining the intracellular GSH homeostasis, stabilizing the cell’s antioxidant capacity, and inhibiting the ferroptosis-like cell death induced by CoNPs.Besides, Since the intracellular and extracellular cobalt concentration of the CoNPs–treated group increased significantly, and ALA has a significant antagonistic effect on this. Combined with the TEM image display, it can be speculated that ALA can inhibit the absorption of CoNPs by cells or promote the excretion of CoNPs (Fig. 7e), thereby decreasing the concentration of Co in cells and alleviate the damage of CoNPs to cells.

## Conclusion

Cobalt is the key element in the cemented carbide industry and one of the major components of human metal implants. CoNPs is not only an important cause of cobalt toxicity but also one of the current hotspots in the field of nanomedicine research. The cobalt exposure to humans and the related toxicity problems deserve to be studied. Previous studies have only focused on the oxidative stress mechanism of CoNPs. Based on oxidative stress, we further found that CoNPs can induce ferroptosis-like cell death in Balb/3T3 cells, while ALA exhibited a protective effect. The present study provides the basis for further investigation of the exact molecular mechanism and related signal pathways of ferroptosis-like cell death induced by CoNPs in Balb/3T3 cells.

## Materials and methods

### Reagent and chemicals

CoNPs (< 50 nm, #7440-48-4), alpha-lipoic acid (#1077-28-7), and dichlorofluorescin diacetate (DCFH-DA) (#4091-99-0) were purchased from *Sigma-Aldrich* (St. Louis, MO). Dulbecco’s modified Eagle's medium (DMEM), fetal bovine serum (FBS), penicillin/streptomycin, and trypsin–EDTA were procured from Gibco (Life Technologies, Paisley, UK). CCK-8 kits were obtained from Dojindo (Kumamoto, Japan). Annexin V-FITC (#AP101-100-AVF), Calcein AM/PI (#C2013FT& ST511) was purchased from Multi Sciences (Hanzhou, China). DMSO, GSH, and GSSG Assay Kit (#S0053) were procured from Beyotime (Shanghai, China). Iron Assay Kit (#ab83366), Anti-GPx4 antibody (#ab125066), Goat anti-rabbit IgG H&L (HRP) (ab205718), Goat anti-mouse IgG H&L (HRP) (ab205719), and β-actin antibody were obtained from Abcam Technology (Cambridge, UK).

### Cell culture and preparation of stock solutions of CoNPs/ALA

Balb/3T3 cell line was procured from the Type Culture Collection of the Chinese Academy of Sciences (Shanghai, China). The cells were cultured in a DMEM medium containing 10% FBS, 50 U/ml penicillin, and 50 mg/ml streptomycin at 37 °C in the presence of 5% CO_2_. The cells were differentiated up to 6 days, and the cell medium of differentiating cells was replaced after every 2 days to ensure the availability of optimal nutrients and differentiation factors for the growth of cells. The cells were sub-cultured after every 3 to 4 days using 0.25% trypsin/1 mM EDTA solution and TNS.

The CoNPs were weighed the day before the experiment, sterilized at 180 °C for 4 h, and suspended in ultrapure water to prepare a stock solution of 6 mM. The freshly prepared stock solution was then ultrasonicated at room temperature at 40 W for 30 min to minimize the particle aggregation and diluted with complete culture medium to achieve the required concentrations. The ultrasonicated fresh stock solution was continuously oscillated using a vortex shaker before adding to the sample well. The solution was distributed to various sample wells within 10 min because of the rapid precipitation of CoNPs (Additional file 1: Figures S1 and S2). Alpha-lipoic acid of appropriate concentration was dissolved in DMSO. The final working concentration of DMSO was controlled below 0.1% to avoid its cytotoxicity.

### CCK-8 cell viability assay

The cell viability assay was performed by employing the Cell Counting Kit-8 (CCK-8). The cells (1.5 × 10^4^ cells/well) were seeded into 96-well plates and adhered for about 8 h before being exposed toward different concentrations of CoNPs (20–800 μM) for 8–24 h at 37 °C to explore the IC50 value. The cells cultured with DMEM media without any CoNPs treatment served as the control group. Further, the cells were incubated with ALA of various concentrations (50–1200 μM) for 24 h to evaluate its cytotoxicity. To investigate the effects of ALA on the viability of Balb/3T3 cells exposed to CoNPs, the cells were first exposed to 400 μM (IC50) of CoNPs and subsequently incubated with ALA (50–600 μM) for 24 h. After that, CCK-8 solution (10 μl) was added to each well of the plate for 1 h at 37 °C before the detection. The CCK-8 formazan product was measured at 450 nm using a microplate reader. The cell viability ratio was expressed as a percentage of the control experiment. Each experimental condition was repeated in triplicate.

### Cell staining to observe live and dead cells

Cells were seeded in a 6-well plate and treated in the same manner as before. After 24 h of exposure, aspirate the medium, add Calcein AM/PI stain and incubate at 37 °C for 30 min. Observe the number of live and dead cells with a fluorescent inverted microscope(LEICA DMi8).

### Detection of intracellular iron ion concentration

The cells were seeded into a 6-well tissue culture plate. When the cells were in the logarithmic growth phase, the cells were pre-treated with ALA (200 μM) for 3 h, followed by incubation with CoNPs (400 μM) for a total of 24 h. The cells were collected, washed with 1 ml phosphate-buffered saline (PBS), and centrifuged for 5 min (1000 rpm) to remove the supernatant. The cells were then resuspended in 0.5 ml PBS, and the cell concentration was calculated using a cell counter. Next, 1 × 10^6^ cells were again centrifuged at 1000 rpm for 5 min, and all the supernatant was carefully aspirated. After that, 500 μl of lysis buffer was added to the cells and place on a shaker for 2 h to lyse them. The Iron Assay Kit was then employed according to the manufacturer's instructions to detect the intracellular iron(II) ion concentration and total iron by spectrophotometry.

### Detection of intracellular and extracellular cobalt concentration

Cells were seeded in a 6-well plate and incubated. When the cells were in the logarithmic growth phase, aspirate the medium, 2 ml of medium containing CoNPs (400 μM), and ALA (200 μM) were added to each well. After 24 h of incubation, 1 ml of the upper-medium was drawn before collecting the cells to measure the concentration of cobalt in the extracellular solution. Then, the medium was removed, and cells were washed with PBS 3 times, detached, and collected with centrifugation at 1000 rpm for 5 min. 2 mL of PBS was used to re-suspend the cells. After 5 min, 10 uL of supernatant was extracted to check with a microscope to confirm there are no CoNPs in the solution. Next, 1 × 106 cells were again centrifuged at 1000 rpm for 5 min. After removing the supernatant, 500 μL of lysis buffer was added, followed by centrifugation at 12,000 rpm for 2 h. Finally, the supernatant was used to measure the cobalt concentration with an inductively coupled plasma mass spectrometer (ICP-MS, PerkinElmer NexION 350).

### Detection of cell necrosis and apoptosis ratio

To compare the ratio of apoptosis and necrosis among the cells in each group, the Annexin V-FITC assay kit was conducted according to the manufacturer's protocol. Briefly, after incubating the cells with respective treatments for a total of 24 has previously been described, they were carefully washed with PBS solution and collected. The cells were resuspended gently with PBS and counted using a cell counter. Next, 1 × 10^6^ cells were centrifuged at 1000*g* for 5 min, and the supernatant was discarded. The cell pellet was incubated with Annexin V-FITC and propidium iodide staining solution for 10–20 min at room temperature (20–25 °C) in the dark and then placed in an ice bath. A flow cytometer (Becton Dickinson flow cytometer) was employed for the detection of cell necrosis and apoptosis. The ratios of apoptosis and necrosis were calculated separately.

### Determination of intracellular ROS

To assess the ROS generation in cells exposed to target concentrations of CoNPs and the protective effects of ALA, spectrofluorometry, and fluorescent microscopy were employed. For spectrofluorometry analysis, the cells (1 × 10^5^/well) were seeded into 96-well tissue culture plates. After 8 h of adherence, the cells were incubated with CoNPs (400 μM) and ALA (200 μM) for 24 h. Following staining with DCFDA for 30 min, the cells were washed twice by 200 μl of PBS. The fluorescent intensity of the suspension was measured using a multi-well microplate reader (Varioskan LUX, Thermo Fisher, USA) at excitation and emission wavelengths of 485 nm and 525 nm, respectively. Values were expressed as the percentage of fluorescent intensity relative to the control wells. The intracellular fluorescence of cells was also measured using an upright fluorescent microscope equipped with a CCD camera (Nikon, Japan).

### Lipid peroxidation (MDA) assay

The cells were treated as described earlier followed by centrifugation at 8000×*g* for 4 min to obtain the supernatant cell lysate, which was used for measuring lipid peroxidation (MDA) according to the manufacturer’s protocol. In brief, the samples and standards were incubated with a TBA solution at 100 °C for 30 min. The mixture was cooled in an ice bath, followed by centrifugation at room temperature for 10 min. Next, 200 µl of the supernatant was placed into a 96-well tissue culture plate, and the absorbance of each sample was measured at 450 nm, 532 nm, and 600 nm. The calculation was performed using following formula: ΔA450 = A450_measurement-_A450_blank_, ΔA532 = A532_measurement_-A532_blank_, ΔA600 = A600 _measurement_-A600_blank_.

### GSH/GSSH measurement

The cells were exposed to CoNPs (400 μM) and ALA (200 μM) for 24 h. The blank group, positive control group (CoNPs, 400 μM), and a negative control group (ALA, 200 μM) were also established at the same time. After the exposure, the cells were washed with PBS twice, scraped off, suspended in PBS, and centrifuged at 1000×*g*. The cell pellet was then homogenized in 5% 5-sulfosalicylic acid. Next, the suspension was lysed by freezing and thawing twice after 5 min centrifuged at 10,000×*g* for 10 min. Intracellular total GSH and GSSG concentrations were measured according to the supplier's protocol.

### Western blot analysis to check the level of GPx4

The cells were treated as described above, collected, and washed twice with cold PBS, followed by lysing with RIPA buffer containing 1 mM phenyl sulfonyl fluoride on ice for around 30 min. The lysed cells were centrifuged at 12,000×*g* for 15 min to obtain the supernatant cell lysate, and protein concentrations were measured using the bicinchoninic acid (BCA) assay kit. The concentrated protein samples were subjected to 15% SDS polyacrylamide gel electrophoresis and transferred to a polyvinylidene difluoride (PVDF) membrane by a transfer apparatus at 300 mA for 1 h. The membranes were then blocked and incubated with primary rabbit antibody specific for GPx4 at 4 °C for overnight. After carefully washing with TBST, the membranes were incubated with HRP-conjugated secondary antibody for 2 h at room temperature. After that, the PVDF membranes were washed with TBST for four times, and the blot was developed using ECL reagent according to the manufacturer’s protocol.

### Observation of cell structure changes by transmission electron microscope

The cells were treated as described above, harvested and washed twice with PBS, followed by centrifugation at 10,000×*g* for 5 min. Next, the excess PBS was aspirated, and glutaraldehyde was added for fixation. Finally, the cells were treated with osmic acid, ethanol, acetone, and other steps. The changes of cells were observed with a transmission electron microscope.

### Statistical analysis

All the experiments were performed in triplicates, and the data were expressed as mean ± standard deviation (SD). All statistical analyses were performed by GraphPad Prism 8 software (La Jolla, California, USA) employing a one-way analysis of variance, followed by Dunnett’s test to evaluate the significance relative to the control experiment. Differences between groups were considered significant if the p-value is less than 0.05.

## Supplementary information

**Additional file 1: Figure S1.** Distribution of CoNPs in the well. CoNPs were treated as described above methods, and then quickly added to each well. Microscopic observations showed that the CoNPs were evenly distributed at the bottom of the wells and were sand-like particles, which proved that the amount of CoNPs in each well was accurate. a) the distribution of CoNPs in blank well(20X); b) CoNPs are evenly distributed in the cell without agglomerating into larger clusters(20X). **Figure S2.** TEM image of CoNPs in the cells. Cobalt nanoparticles are distributed in clusters in cells, and the diameter of the cobalt nanoparticles is less than 5 0nM, which is round or elliptical. **Figure S3. **Calcein AM/PI staining image (×20).The results showed that 400 μM CoNPs caused significant changes in cell viability, smaller cell size, and more dead cells (red) than live cells (green). ALA significantly reduces cell mortality and maintains cell morphology and vitality. **Figure S4. **CoNPs accumulate in clusters in cells. CoNPs are encapsulated into multiple vesicles in the cell and can be discharged out of the cell through the vesicles, indicating that vesicular transport may participate in the intracellular transport of cobalt nanometers. **Table S1.** The effects of ALA and CoNPs on intracellular GSH and GSSG.

## Data Availability

We ensure the authenticity and repeatability of the data obtained by this research. The datasets used and/or analyzed during the current study are available from the corresponding author. All data generated or analyzed during this study are included in this published article. For more information please email our Research Data Team(liufanntu19575@163.com).
