# Supplementary material for: Alpha lipoic acid antagonizes cytotoxicity of cobalt nanoparticles by inhibiting ferroptosis-like cell death
Source: J Nanobiotechnology. 2020 Oct 2;18:141. doi: 10.1186/s12951-020-00700-8 (PMC7532644; doi:10.1186/s12951-020-00700-8)
Supplement: Supplementary file 1 — Additional file 1: Figure S1. Distribution of CoNPs in the well. CoNPs were treated as described above methods, and then quickly added to each well. Microscopic observations showed that the CoNPs were evenly distributed at the bottom of the wells and were sand-like particles, which proved that the amount of CoNPs in each well was accurate. a) the distribution of CoNPs in blank well(20X); b) CoNPs are evenly distributed in the cell without agglomerating into larger clusters(20X). Figure S2. TEM image of CoNPs in the cells. Cobalt nanoparticles are distributed in clusters in cells, and the diameter of the cobalt nanoparticles is less than 5 0nM, which is round or elliptical. Figure S3. Calcein AM/PI staining image (×20).The results showed that 400 μM CoNPs caused significant changes in cell viability, smaller cell size, and more dead cells (red) than live cells (green). ALA significantly reduces cell mortality and maintains cell morphology and vitality. Figure S4. CoNPs accumulate in clusters in cells. CoNPs are encapsulated into multiple vesicles in the cell and can be discharged out of the cell through the vesicles, indicating that vesicular transport may participate in the intracellular transport of cobalt nanometers. Table S1. The effects of ALA and CoNPs on intracellular GSH and GSSG. [file 12951_2020_700_MOESM1_ESM.docx]

**Additional file**

**Alpha lipoic acid antagonizes cytotoxicity of cobalt nanoparticles by inhibiting ferroptosis-like cell death**

Yake Liu ^a,b, 1^, Wenfeng Zhu ^a, 1,^ Dalong Ni^c^, Zihua Zhou ^b^, Jin-hua Gu^d^, Weinan Zhang ^a,b^, Huanjian Sun ^c^, Fan Liu ^a, *^

a Department of Orthopaedics, Affiliated Hospital of Nantong University, Nantong, Jiangsu Province, China;

b Orthopaedic Laboratory, Affiliated Hospital of Nantong University, Nantong, Jiangsu Province, China;

c Department of Radiology, University of Wisconsin-Madison, 11111 Highland Avenue, Madison, Wisconsin. 53705;

d Department of clinical pharmacy, Affiliated Maternity and Child Health Care Hospital of Nantong University, Nantong, Jiangsu Province, China;

e Department of Orthopaedics, The Sixth Affiliated Hospital of Nantong University, Yancheng, Jiangsu Province, China.

* Corresponding author. Department of Orthopaedic Surgery, Affiliated Hospital of Nantong University, 20 Xisi Road, Nantong, Jiangsu 226001, China.

E-mail address: liufanntu19575@163.com (Fan Liu)

1 Yake Liu and Wenfeng Zhu contributed equally to this work.


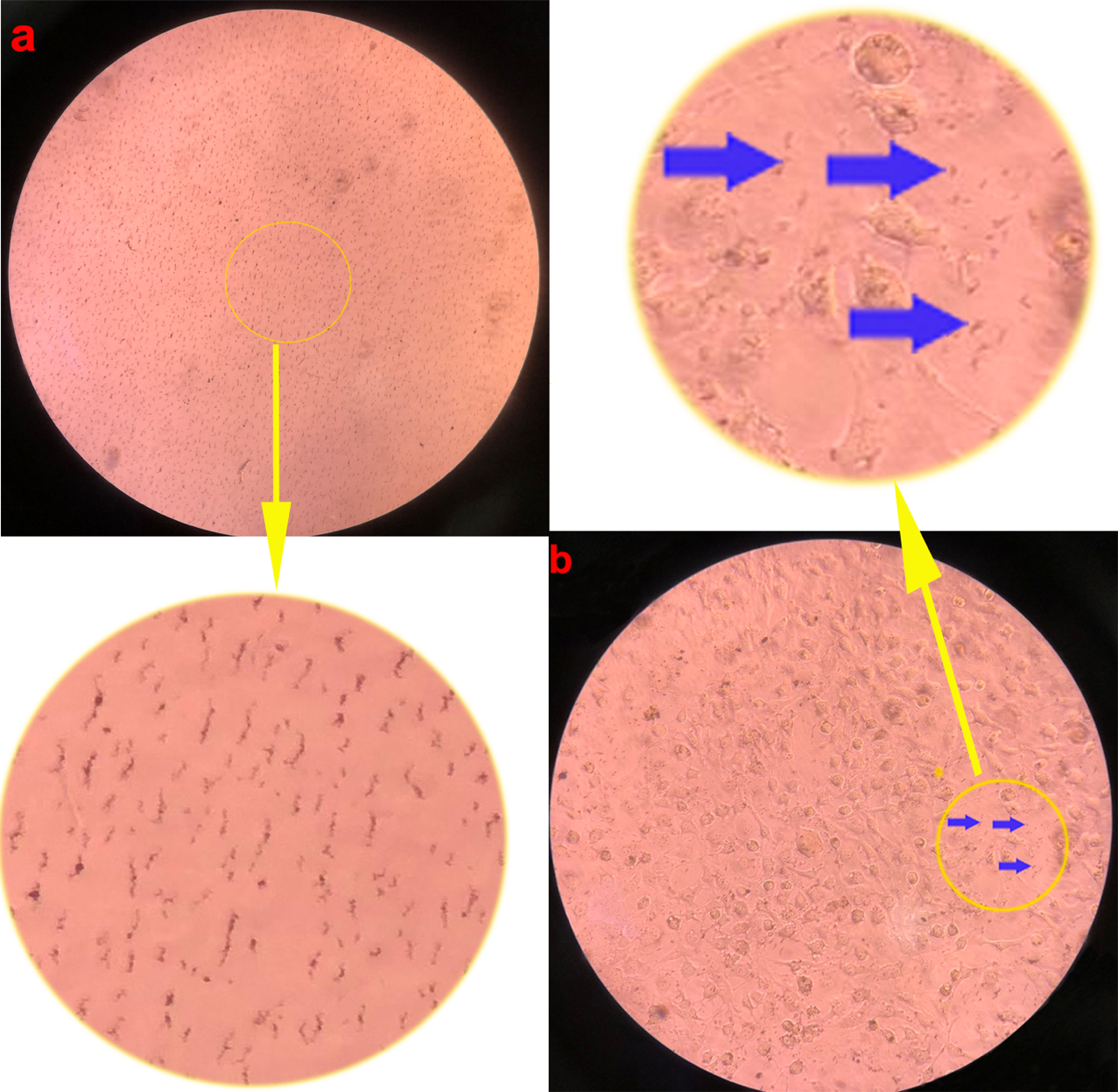
**Figure S1. Distribution of CoNPs in the well.** CoNPs were treated as described above methods, and then quickly added to each well. Microscopic observations showed that the CoNPs were evenly distributed at the bottom of the wells and were sand-like particles, which proved that the amount of CoNPs in each well was accurate. a) the distribution of CoNPs in blank well(20X); b) CoNPs are evenly distributed in the cell without agglomerating into larger clusters(20X).


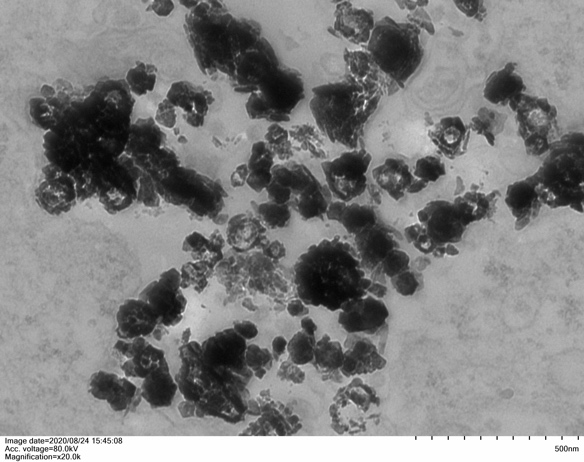

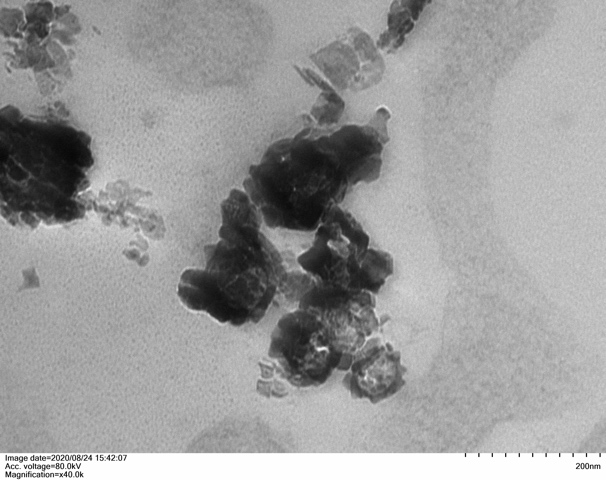


**Figure S2. TEM image of CoNPs in the cells.** Cobalt nanoparticles are distributed in clusters in cells, and the diameter of the cobalt nanoparticles is less than 50nM, which is round or elliptical.


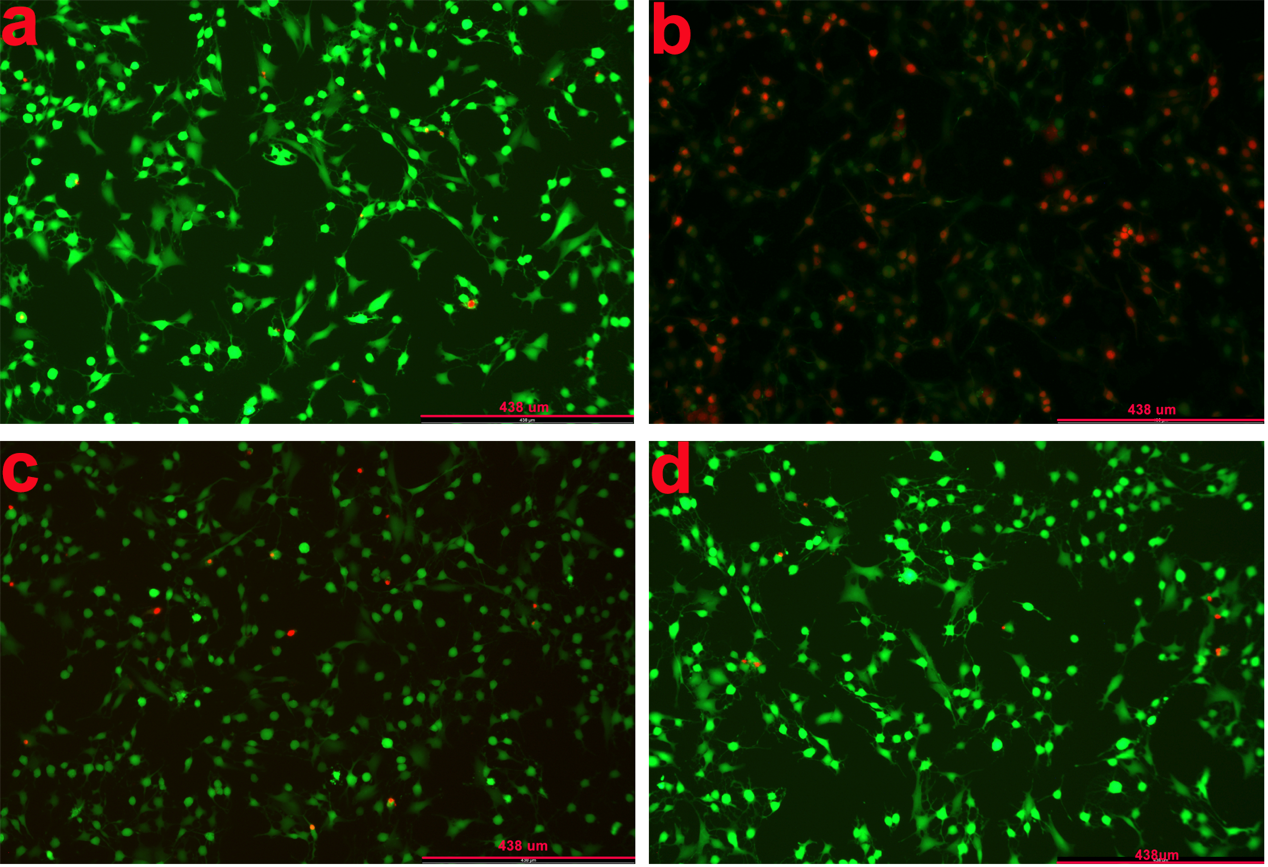


**Figure S3. Calcein AM/PI staining image（×20）.**The results showed that 400μM CoNPs caused significant changes in cell viability, smaller cell size, and more dead cells (red) than live cells (green). ALA significantly reduces cell mortality and maintains cell morphology and vitality.


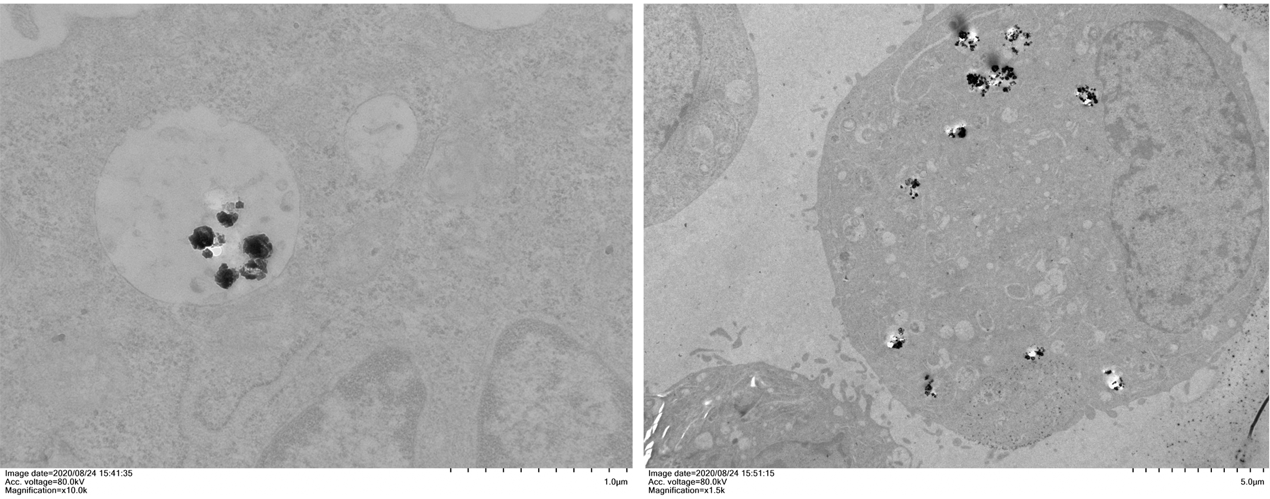


**Figure S4. CoNPs accumulate in clusters in cells.** CoNPs are encapsulated into multiple vesicles in the cell and can be discharged out of the cell through the vesicles, indicating that vesicular transport may participate in the intracellular transport of cobalt nanometers.

**Table S1. The effects of ALA and CoNPs on intracellular GSH and GSSG.**

| Group | T-GSH  (nmol/mg) | GSH  (nmol/mg) | GSSG  (nmol/mg) | GSH/GSSG |
| --- | --- | --- | --- | --- |
| Control | 57.1 ± 9.1 | 52.6 ± 11.3 | 2.4 ± 1.1 | 22.1 ± 7.2 |
| CoNPs | 32.2 ± 10.2 | 17.8 ± 9.4 | 10.7 ± 3.1 | 2.4 ± 1.6 |
| CoNPs+ALA | 54.6 ± 7.3 | 46.8 ± 10.8 | 5.3 ± 1.9 | 9.3 ± 7.6 |
| ALA | 68.4 ± 13.4 | 64.9 ± 12.8 | 2.1 ± 0.9 | 31.8 ± 6.9 |

Compared to the control group, CoNPs can significantly reduce the intracellular level of total GSH, GSH, and GSG/GSSH ratio. The cells in the co-exposure group (CoNPs+ALA) did not exhibit any decrease in the level of total GSH, GSH, and GSSG.
